# Using cognitive theory to facilitate medical education

**DOI:** 10.1186/1472-6920-14-79

**Published:** 2014-04-14

**Authors:** Yu Qi Qiao, Jun Shen, Xiao Liang, Song Ding, Fang Yuan Chen, Li Shao, Qing Zheng, Zhi Hua Ran

**Affiliations:** 1 Division of Gastroenterology and Hepatology, Ren Ji Hospital, School of Medicine, Shanghai Jiao Tong University, Shanghai Institute of Digestive Disease, 160# Pu Jian Road, Shanghai, China 200127; 2 Division of Cardiology, Ren Ji Hospital, School of Medicine, Shanghai Jiao Tong University, 160# Pu Jian Road, Shanghai, China 200127; 3 Division of Hematology, Ren Ji Hospital, School of Medicine, Shanghai Jiao Tong University, 160# Pu Jian Road, Shanghai, China 200127; 4 Internal medicine teaching and research office, Ren Ji Hospital, School of Medicine, Shanghai Jiao Tong University, 160# Pu Jian Road, Shanghai, China 200127; 5 Ren Ji Clinical Medical College, Ren Ji Hospital, School of Medicine, Shanghai Jiao Tong University, 160# Pu Jian Road, Shanghai, China 200127

**Keywords:** Working memory, Cognitive load theory, Schemata, Clinical reasoning, Worked example, Problem based learning, Clinical presentation curriculum

## Abstract

**Background:**

Educators continue to search for better strategies for medical education. Although the unifying theme of reforms was “increasing interest in, attention to, and understanding of the knowledge base structures”, it is difficult to achieve all these aspects via a single type of instruction.

**Methods:**

We used related key words to search in Google Scholar and Pubmed. Related search results on this topic were selected for discussion.

**Results:**

Despite the range of different methods used in medical education, students are still required to memorize much of what they are taught, especially for the basic sciences. Subjects like anatomy and pathology carry a high intrinsic cognitive load mainly because of the large volume of information that must be retained. For these subjects, decreasing cognitive load is not feasible and memorizing appears to be the only strategy, yet the cognitive load makes learning a challenge for many students. Cognitive load is further increased when inappropriate use of educational methods occurs, e.g., in problem based learning which demands clinical reasoning, a high level and complex cognitive skill. It is widely known that experts are more skilled at clinical reasoning than novices because of their accumulated experiences. These experiences are based on the formation of cognitive schemata. In this paper we describe the use of cognitive schemata, developed by experts as worked examples to facilitate medical students’ learning and to promote their clinical reasoning.

**Conclusion:**

We suggest that cognitive load theory can provide a useful framework for understanding the challenges and successes associated with education of medical professionals.

## Background

Medical education has changed significantly over time. The changes are linked not only to changes in educational technology and the advancement of medical knowledge, but also in educational concepts and curricula. From the 1940s, key principles and practices of the discipline-based model were questioned by educators. In 1944, Goodenough advocated for a reduction in basic science detail which seemed to “clutter up the student’s mind and deaden interest” [1]. Sinclair [2] noted that medical students are often attracted to medicine by a sense of idealism, but the premedical, preclinical and clinical sequence of instruction discourages them.

An early wave of innovation saw a revised curriculum which focused on organ systems. This may be one of the earliest examples of an attempt to better integrate the curriculum. Two different models followed which then catalyzed significant changes in medical curricula. One is problem based learning (PBL), which has been in use since 1971 and still remains popular in many medical schools. The other is the clinical presentation (CP) based model, which has been used since 1991.

Educators continue to search for better strategies for medical education. Papa et al. [3] detail the unifying theme of reforms: increasing interest in, attention to, and understanding of the knowledge base structures and cognitive processes that characterize and distinguish medical experts and novices. There are three main aspects to this theme: (1) more attractive courses; (2) well organized knowledge; and (3) proper guidance for different students. Although these aspects are relatively independent, they are often confused by educators. Attractive courses are not necessarily effective, and effective training is not always interesting. It is difficult to achieve all these aspects via a single type of instruction.

In this paper, we aim to answer several questions: (1) What is the basic step in medical learning, to think or to memorize? (2) Why do some curricula seem attractive, but prove not to be effective? (3) What are the differences between novices and experts? (4) How can novices become experts, and how best can novices be given instruction? Answers to these questions are complex and widely debated. However, we argue that cognitive load theory proposed by Sweller et al. [4-8] holds answers to these questions. Cognitive load theory is based on human cognitive architecture and assumes a limited working memory capacity. We argue that when designing instruction, it must be taken into more explicit consideration.

## Methods

PBL has been shown to be less effective than expected in teaching of internal medicine [9]. We aimed to determine the reasons for this. As a first step, key words linked to medical education were selected and used in a literature search. These included ‘medical education’, ‘curriculum’, ‘problem based learning’, and ‘clinical reasoning.’ ‘Advantage’, ‘disadvantage’ or ‘limit’ were also used when needed. For the second step, we added several other key words, closely related to cognitive load theory. They included ‘cognitive load’, ‘schema’, ‘worked examples’ and ‘working memory.’ We used these key words to search in Google Scholar (http://scholar.google.com) and Pubmed (http://www.ncbi.nlm.nih.gov/pubmed/). Related search results on this topic were selected for discussion. The full text of articles was acquired from the open internet proxy service of Shanghai Jiao Tong University and the library of the School of Medicine, Shanghai Jiao Tong University. The research protocol was approved by the Ethics Committee of Ren Ji Hospital, School of Medicine, Shanghai Jiao Tong University.

## Results and discussion

### Memorizing: a basic step in learning

What is the most basic step in medical learning? To make students “thinkers” rather than “memorizers” was the hallmark of the discipline-based curricular movement, which dates back to the late 19^th^ century. In 1898, Sternberg echoed the importance of thinking in his American Medical Association (AMA) presidential address [3]. Over the next hundred years, educators continually made efforts to support this view and the importance of thinking over memorizing. However, we contend that to be a thinker, one first needs to be a memorizer. Although it may not follow that a good thinker should be a good memorizer, a good thinker most likely needs to draw on a wide and deep reservoir of memorized knowledge. In Sweller’s words, the more sophisticated and knowledgeable the learner, the more complex will be the elements he or she is dealing with [10]. An element is defined here as material to be learned.

The definitions of short-term memory (STM) and long-term memory (LTM) indicate the different time spans of the information retention associated with each. STM retains information for just a few seconds. LTM can hold information for several days or decades. Working memory (WM) stores task-relevant information and involves “the temporary storage of information that is being processed in any of a range of cognitive tasks” [11,12]. Because of temporary storage, WM was once considered as a function of STM. Now, many researchers believe STM is in fact a component of WM, and there are other mechanisms based on skilled use of storage in LTM. Ericsson and Delaney used the term “long-term working memory (LTWM)” to refer to information that is stored in stable form, but could be retrieved temporarily by means of cues in STM [13]. Baddeley considered this as an interactive mode between LTM and WM rather than a new type of WM per se [14].

As LTM has been demonstrated as an important source of WM, knowledge in LTM should not be neglected. In medical education, acquisition of declarative knowledge depends on memory, especially LTM, and LTM can also directly affect actions [14]. Procedural or skills based knowledge should be retrieved from LTM when needed [15]. Problem-solving performance is dependent on knowledge of specific areas, so clinical reasoning ability cannot be separated from LTM. As Clark et al. [16] described, novices can attend the problem-solving process, but they may learn almost nothing. Memorizing should be regarded as a first basic step in learning as it gives a firm foundation for further learning and practice. However, promoting memorizing remains a challenging task.

### Cognitive load theory: a framework for proper instruction

WM capacity (WMC) is limited to holding approximately seven (plus or minus two) elements (or chunks) when processing information [17]. Studies have indicated that WMC limits students’ performance in science learning and problem solving [18-20]. Similarly, cognitive load theory supports the idea that WM limits the amount of information an individual can process [5,21]. The theory suggests that there are two main types of cognitive load, intrinsic and extraneous load. Germane load can further be considered as a supplementary type.

Intrinsic load is based on the interactivity of elements in learning materials. As it refers to the nature of learning materials, it is constant for a given area. In some subjects, interactions between many elements must be learned, and the intrinsic cognitive load will be high [5,21,22]. Intrinsic cognitive load depends on a learner’s prior knowledge [23]. When many new elements appear together in learning materials, novices may experience learning difficulties even if the interactivity of elements is low. The learning process may involve combining single elements into chunks. These chunks are defined as cognitive schemata and can be stored in LTM [24]. Another way of viewing cognitive schemata is to see them as highly organized information or knowledge. Over time, as prior knowledge forms into schemata, expertise increases, and the intrinsic load can be reduced.

Total cognitive load, the working memory load experienced by learners, is not only based on the intrinsic load, but also linked to the teaching methods employed. When these do not promote learning, they can be defined as extraneous cognitive load. Extraneous cognitive load arises from inappropriate guidance that cannot contribute to the construction of cognitive schemata [24]. In contrast with extraneous cognitive load, germane load arises from the process of forming cognitive schemata. Germane load was added to the cognitive load framework as a supplement [4]. In recent explanations of the theory, germane load was termed as germane resources and closely related to intrinsic load [5,6]. As there is no necessity to include germane load in specific empirical results, Kalyuga redefined it as working memory resources dealing with intrinsic load [25].

In medical learning, considering only the large volume of information, the challenges associated with learning can be very high. For clinical reasoning education, the cognitive load becomes higher because of the high interactivity of elements. The large amount of information and high intrinsic cognitive load can explain why medical learning is most often a challenge. Learning processes could be affected when the cognitive load exceeds the limit of WMC [26]. Medical students have to take considerable time in memorizing and understanding the unrelated learning materials. One solution is to decrease the extraneous cognitive load in learning materials [26].

Moving to our second question: Why do some curricula seem attractive, but not effective? High intrinsic cognitive load causes difficulties when studying medicine because of the large volume of information. When inappropriate teaching methods are used (e.g., guidance integrated with different disciplines or problem based exploration) this will serve to even further increase the extraneous cognitive load without decreasing the burden of intrinsic load.

Although cognitive load theory has not always been explicitly considered, there is evidence for its effectiveness. In the 1950s, the Western Reserve School of Medicine initiated the first organ-system curriculum [27]. Educators aimed to help students integrate knowledge of system-oriented functions and malfunctions. This reform failed because while it appeared to integrate basic science with clinical knowledge, learners struggled to achieve this integration. As the amount of information was not reduced and interacting elements were added to the curriculum, extraneous cognitive load became untenably high, especially for junior medical students. Students in the early years of their degree programs have limited background knowledge of medicine, and are not able to effectively chunk the large number of elements into schemata. The cognitive load of the organ-system framework most likely exceeded the students’ WMC. The demands of that process may be viewed as analogous to expecting novice foreign language learners to readily master grammar.

PBL focuses on clinical cases and is widely used in medical education. There has been considerable debate about PBL, and attitudes towards the approach vary considerably. Literature suggests that it can be used as an effective approach, but this is not always the case. Patel et al. found that PBL students were less accurate in diagnosis and made more conceptual errors than their more traditionally trained counterparts [28]. Albanese et al. carried out a meta-analysis focusing on PBL and its effectiveness [29]. They found that PBL students spent more time studying, but that their basic science exam scores were lower than students who were being trained using other methods. Although PBL students achieved better scores for their clinical performance, they were found to order significantly more unnecessary tests for their patient, which means a higher cost with less benefit. In Berkson’s meta-analysis, similar conclusions were reached [30]. Colliver reviewed PBL literature and noted no convincing evidence that PBL improves students’ knowledge base and clinical performance [31]. While Kirschner debates the effect of PBL [9], Sanson-Fisher et al. highlight a lack of evidence supporting the superiority of PBL over traditional methods [32]. PBL is innovative, but has yet to meet the requirements of evidence-based practice. Sweller et al. emphasize that PBL may teach students how to find information, but it does not reduce the relevant information that is ultimately required to be assimilated [33]. A more recent meta-analysis suggests that unassisted discovery does not benefit learners [34]. Conversely, feedback, worked examples, scaffolding, and elicited explanations might work.

PBL aims to improve the hypothetico-deductive reasoning (HDR) of students. However, HDR brings with it a high cognitive load, which needs some degree of prior background knowledge and some basic skills. Students may face the same challenges in PBL as in the organ-system curriculum. Before acquiring basic knowledge, the cognitive load is high and materials might be hard for students to understand. This can explain why PBL students spend more study hours and make more conceptual errors. Haeri et al. did not recommend widespread use of HDR [35]. Although the full use of PBL is not advocated for medical curricula, it should be noted that this does not mean that PBL is totally outdated or inappropriate. It does have some uses in medical education, especially if understood in light of the cognitive load theory, a point to which we return later.

### Schemata: distinguishing experts from novices

Inappropriate instruction cannot promote learning, especially in novices. Before giving proper instruction, we consider the differences between novices and experts. While a person may have the capability to memorize Shakespeare’s dramas, it does not mean that s/he would then have the writing skills of Shakespeare! Could one learn more when memorizing more? Although this seems logical, it may not be accurate. A study of physicians has shown a positive relationship between memory for numerical laboratory data and expertise [36]. However, when participants were informed at the outset that a memory test would be given, the difference between experts and students was no longer reliable. This retrieval task showed that there is no significant memory difference between experts and students, but experts can memorize related information simultaneously. While storing information in LTM is the basic step of learning, Paas et al. suggested two other critical mechanisms: schemata acquisition and the transfer of learned procedures from controlled to automatic processing [37]. Evidence showed that domain specific knowledge in the form of schemata is the primary factor distinguishing experts from novices in learning and problem-solving skill [21].

Schemata consist of highly organized knowledge and information. According to Elstein, knowledge organization and schemata acquisition are important in developing expertise [38]. For example, experiments suggest that chess experts can remember 50,000 game positions. This superior memory is thought to be mediated by the familiar and meaningful configurations of the chess pieces [39]. A chess master stores a large amount of information regarding the specific patterns of chess pieces in LTM, and memory representations allow for the rapid recognition of patterns in a presented chess position. Cooke et al. contended that skilled chess players encode chess positions in terms of high-level descriptions of their structure [40].

The research on chess players suggests two aspects of organized knowledge in LTM: (1) Using organized knowledge can increase the speed of retrieval from LTM; (2) After a training period, a large amount of organized knowledge relating to a specific area can be stored in LTM. New information can be organized more quickly by experts in a specific field. A schema is a cognitive construct that organizes the elements of information according to how they will be processed [21]. In Sweller’s cognitive load theory, schemata could reduce elements’ interactivity, and thereby reduce the intrinsic cognitive load. According to Ericsson et al., the bottleneck for retrieval from LTM is the lack of retrieval cues that relate to the desired item stored in LTM [13]. Schemata represent a higher level of organized knowledge than a simple collection of lower-level components [41]. Although organized knowledge cannot increase the retrieval cues, it can reduce the need for such cues. Schemata that contain automated procedural knowledge, can be considered as a whole to be retrieved together. Learners with higher levels of knowledge can retrieve appropriate schemata and generate more appropriate solutions.

Now we can also explain why senior doctors are better at clinical reasoning than novices. Clinical reasoning is complex and requires breadth of knowledge, knowledge organization and retrieval ability, and that each of these aspects work together. Doctors will always require a broad perspective of organized knowledge for their reasoning. Schmidt describes how experts may have access to extensive case knowledge, but this knowledge remains ‘encapsulated’ until needed [42]. Rikers et al. showed that encapsulated knowledge plays a very important role in specialists’ clinical reasoning [43] and that this is very different from the clinical reasoning of senior medical students. While senior medical students could use basic scientific knowledge to explain a phenomenon, specialists made more accurate diagnoses even in areas beyond their own specialty.

Norman reviewed research focusing on clinical reasoning [44]. Evidence suggests that expertise is distinguished by acquisition of illness scripts, decision trees, symptoms, disease probabilities, semantic qualifiers and more (or less) basic science. Clinicians appear to move through three different kinds of mental representations, from basic mechanisms of disease to illness scripts to exemplars derived from experience [45]. The developmental theory of medical expertise is based on increasing clinical experience. Kyllonen et al. found that reasoning correlated highly with general knowledge [46]. Experience might be the most significant difference between novices and experts. Norman’s work suggested to us that focusing on clinical experts might be a useful way to promote clinical reasoning. To study from clinical experts is to study their experiences. The increasing store of experiences results in more schemata formation. Merriënboer et al. described learning as the construction and automation of such schemata [8]. A randomized trial by Blissett et al. focused on schema based teaching [47]. It showed such instruction could improve retention of structured knowledge and diagnostic performance among novices. To study clinical experience might be easier and more effective than to study reasoning strategies since there is most likely no one particular reasoning strategy used.

### Proper instruction helps novices become experts

How can we make novices become experts? How can we give proper instruction to novices? In a traditional discipline-based curriculum, students need to memorize a large volume of information presented in no particular order. They have to integrate this information into clinically meaningful knowledge. Educators are always looking for ways to lower the burden of remembering and trying to integrate information into a logical order that is easy for students to learn. Worked examples may be a good method for novices. This involves presentation of a real task process rather than just explaining how it works. When giving worked examples, the experience is contained. Imitation is a good way of learning with the lowest cognitive load. It is reasonable to give an example first and then explain why. Tuovinen et al. found that students with no previous domain familiarity could substantially benefit from worked examples in comparison to exploration [44]. Sweller et al. also demonstrate how worked examples can be an effective way to reduce extraneous cognitive load [4]. They showed worked examples with annotations regarding crucial features were helpful for students in applying schemata in problem-solving [48,49]. A study of novices showed that students who studied applications of Bayes’ theorem in example-example or example-problem conditions performed better than their peers who studied the applications in problem-example or problem-problem conditions [24].

In medical education, it is not easy to form organized knowledge through clinical reasoning strategy training [44]. However, educators at the University of Calgary’s Faculty of Medicine (UCFM) used the idea of worked examples to achieve this. They shared the experience of experts using developed schemes with medical students [3,50]. They assessed the advantages and disadvantages of the former medical curriculum to revise and form a new curriculum. By 1991, the clinical presentation curriculum (CPC) was ready for use and consisted of 120 clinical presentations [50]. Since the aim of learning is to know what to do, each presentation described the appropriate clinical procedures for dealing with particular conditions (e.g., loss of consciousness/syncope). Schemata outlined how experienced physicians differentiated one cause from another. The presentations were developed by medical experts and the structure was based on how real patients present to physicians. Current knowledge, principles of adult learning, clinical problem solving, community demands and curriculum management were taken into consideration for the curriculum structure [50]. These worked examples facilitated the students’ learning.

In 1998, educators from UCFM carried out an analysis of the use of CPC. They found that it generated less stress than other curricula, despite an equivalent workload [51]. In 2009, a comparison of a 3- and 4-year curriculum was made by Lockyer et al. [52]. CPC was applied in a 3-year curriculum at UCFM. The results showed no significant difference between students from UCFM and those following a 4-year curriculum. Students from UCFM were saving about 3 months of medical study time to meet the same standard. Although UCFM have provided the only data based evidence of CPC to date, other researchers have given their support to CPC. Tsai introduced CPC and supported its use in Taiwan [53]. Haeri et al. also recommended CPC as a more appropriate choice than PBL. CPC may offer a preferable way of training, but its focus is on clinical education rather than on preclinical work or basic sciences [35]. Worked examples can foster diagnostic knowledge [54]. van den Berge et al. showed that novices who studied worked examples of electrocardiograms (ECG) performed better on a retention test [55]. Worked examples can also be applied to the acquisition of visual perceptual skills, and have even been used in more complex skills training, such as bronchoscopy and catheter-based cardiovascular interventions [56,57].

Returning to PBL, we note that this approach has been used in a range of different areas. In medical education, PBL is considered an appropriate way to promote HDR abilities and it has been implemented in many curricula [58]. PBL has been widely used as a way to encourage exploration, but many scholars argue that its use in medical education needs reconsideration [9]. Today there are still many PBL curricula used in medical schools around the world. A PBL curriculum will typically give simulated scenarios of real clinical work, with minimal guidance given beforehand. It is a student centered active learning activity which strives to promote self-study [3]. However, there is no evidence to show that PBL students perform better than students following a traditional curriculum. This may be because of the inappropriate implementation of PBL. As PBL is an HDR process with feedback, it has to be supported by sufficient related knowledge. Novices have yet to acquire this knowledge. They lack the knowledge to complete the process. In early stages of medical education, basic science and preclinical courses are the main components of learning. Memorizing knowledge plays an important role at that stage. There is a large amount of new information in anatomy, pathology, physiology and biochemistry. The task is difficult because of the high volume of information [10]. Using PBL at that stage cannot reduce the intrinsic load. HDR with high interactivity of elements might even increase the extraneous cognitive load. This could explain why PBL students achieve lower scores in basic science [29]. For senior medical students, the situation is quite different, especially when they begin their internships. Senior students have more relative knowledge than novices, and they will be exposed to more clinical problems than their junior peers. As formation of schemata (increasing expertise) can reduce the intrinsic cognitive load, partially or minimally guided instruction can be effective for senior students and residents under supervision [16].

## Conclusion

Medical education reform is ongoing. The motivation underpinning this reform has not changed over time. Although different methods have been trialed in medical education, memorizing is still an important step, especially for the basic sciences. According to cognitive load theory, the intrinsic cognitive load of subjects like anatomy or pathology originates mainly from the large volume of new information. Methods to decrease extraneous cognitive load might not be effective in the case of these subjects. To store more information in LTM might be the only feasible solution. Learning challenges will increase when cognitive load is high. Inappropriate use of educational methods can also increase the extraneous cognitive load. When cognitive load exceeds WMC, it will negatively affect the learning processes. The failure of the organ-system curriculum described in this paper gives an example of this negative overloading effect. Similarly, the inappropriate use of PBL will lead to the same outcome. These curricula seem attractive and innovative, but are not effective. Clinical reasoning is a complex process with a high intrinsic cognitive load. Experts are skilled at clinical reasoning when compared with novices. We have emphasized that this is because of their experiences. These experiences are based on the formation of cognitive schemata. We described the application of such schemata where experts have developed worked examples for students to facilitate their study. Ultimately, this may promote clinical reasoning. Although the full use of PBL is not recommended in medical education, it could be effectively used as a supplementary approach with senior students to facilitate their exploration and self-study.

## Competing interests

The authors declare that they have no competing interests.

## Authors’ contributions

QYQ and SJ have made substantial contributions to the conception and design of this paper. QYQ drafted the manuscript. DS and LX supplied the teaching materials of minimal guidance and contributed to the discussion in the article. SL and CFY supplied the materials related to the clinical reasoning courses and also contributed to the discussion. RZH revised the manuscript critically for important intellectual content. ZQ revised the manuscript based on reviewers’ reports. All authors read and approved the final manuscript.

## Pre-publication history

The pre-publication history for this paper can be accessed here:

http://www.biomedcentral.com/1472-6920/14/79/prepub
